# Absorption-Enhancing Effects of Bile Salts

**DOI:** 10.3390/molecules200814451

**Published:** 2015-08-10

**Authors:** Eskandar Moghimipour, Abdulghani Ameri, Somayeh Handali

**Affiliations:** 1 Nanotechnology Research Center, Ahvaz Jundishapur University of Medical Sciences, Ahvaz 61357-33184, Iran; E-Mail: moghimipour_e@ajums.ac.ir; 2 Department of Drug and Food Control, Faculty of Pharmacy, Ahvaz Jundishapur University of Medical Sciences, Ahvaz 61357-33184, Iran; E-Mail: ghaniameri@yahoo.com

**Keywords:** bile salts, drug delivery, absorption enhancers

## Abstract

Bile salts are ionic amphiphilic compounds with a steroid skeleton. Among the most important physiological properties of bile salts are lipid transport by solubilization and transport of some drugs through hydrophobic barriers. Bile salts have been extensively studied to enhance transepithelial permeability for different marker molecules and drugs. They readily agglomerate at concentrations above their critical micelle concentration (CMC). The mechanism of absorption enhancement by bile salts appears to be complex. The aim of the present article was to review bile salt structure and their application as absorption enhancers and the probable mechanism for increasing permeation based on previous studies.

## 1. Introduction

Bile salts are amphipathic steroidal bio-surfactants that are derived from cholesterol in the liver [1,2,3]. The synthesis of bile salts is the major route for elimination of cholesterol from the body [4]. Bile salts are endogenous surfactants which have been employed widely as absorption enhancers to increase drug transport across various biological barriers such as the blood brain barrier, skin, mucosa, cornea, buccal, nasal, pulmonary and intestinal membranes [5,6]. They act as absorption enhancers by increasing the solubility of hydrophobic drugs or by increasing the fluidly of the apical and basolateral membranes [7] and promote the chemical and enzymatic stability of drugs [8].

## 2. Structure and Synthesis

**Figure 1:**
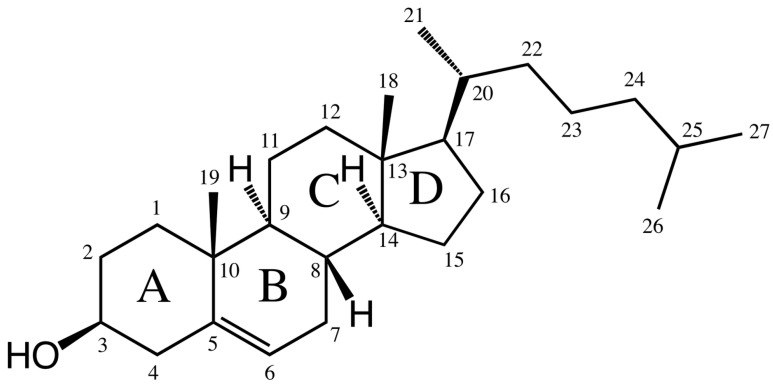
Common chemical structure of bile acids.

Bile acids are ionic amphiphilic compounds with a steroid skeleton. As shown in Figure 1, the structure of bile acids consists of four rings, three six carbon rings (A, B and C) and one five carbon ring (D). With regards to this structure, several bile acids are shown in Table 1 [9,10]. The concave (α) side of the steroid skeleton of bile acid molecules is hydrophilic due to the presence of OH groups, while the convex (β) side with its angular methyl groups is hydrophobic [11]. This structure makes them very different from traditional surfactants, which are often composed of a polar head group and a long non-polar tail [9].

**Scheme 1:**
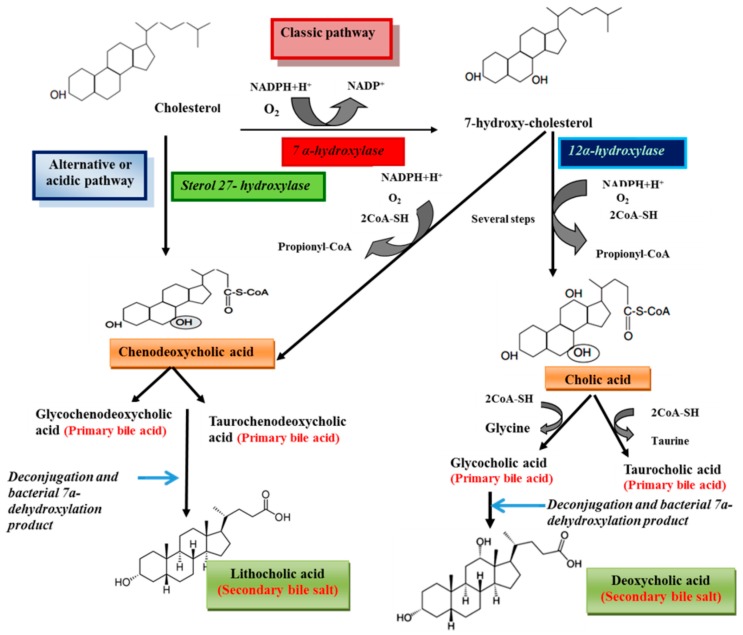
Schematic representation of bile acids biosynthesis from cholesterol.

**Figure 2:**
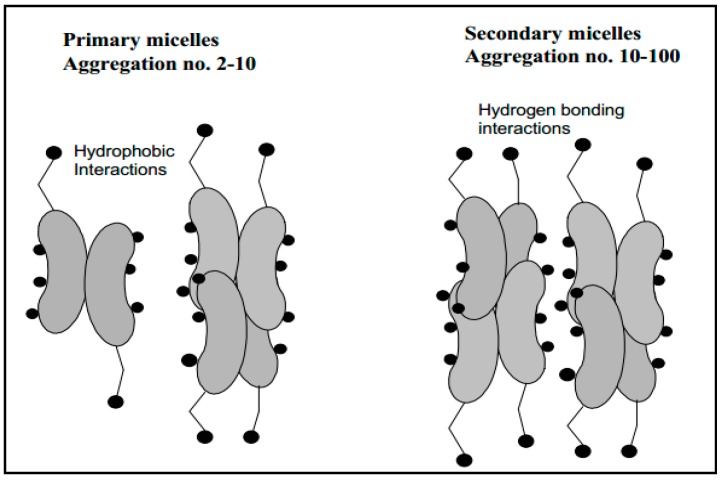
Primary and secondary aggregation model of bile salt micelles [18].

Bile salts are synthesized from cholesterol in the liver [1]. Processes of bile acids biosynthesis from cholesterol are shown in Scheme 1. Cholesterol is eliminated by hepatic biotransformation to bile alcohols or bile acids. These compounds are further biotransformed in the liver by sulfation or amidation and the produced bile salts, are secreted into bile [12]. Bile acids include two groups, primary (cholic acid and chenodeoxycholic acid) and secondary bile acids (deoxycholic acid and lithocholic acid) [9,13]. Primary bile acids are synthesized from cholesterol in the liver *via* different pathways including the classic and alternative ones [14]. The classical pathway (also known as the neutral pathway) is responsible for the large quantities of the bile acids synthesized by the liver [15,16] and only about 10% of bile acids are produced *via* the alternative pathway [17]. The enzyme 7α-hydroxylase, a cytochrome P450 enzyme which converts cholesterol to 7α-hydroxycholesterol, is the main enzyme in the classic pathway. 7α-Hydroxycholesterol is converted to chenodeoxycholic acid or to cholic acid by a pathway that is catalyzed by 12α-hydroxylase [14]. The reaction catalyzed by 7α-hydroxylase is the rate limiting step in bile acid synthesis [18]. The alternative or acidic pathway which generates chenodeoxycholic acid, involves hydroxylation of cholesterol at the 27 position by the mitochondrial enzyme sterol 27-hydroxylase. The acidic pathway can account for 25% and 9% of total bile acid synthesis in mice and humans, respectively [14]. Prior to secretion, bile acids are conjugated with taurine or glycine [14]. Conjugation of bile acids to glycine and taurine reduces the hydrophobicity of bile acids [19,20]. In human bile, the concentration of glycine conjugates (75%) is much higher than taurine (25%) [18]. The primary bile acids are converted to the secondary bile acids following intestinal bacterial hydrolysis of the amide backbone of conjugated bile acids [9]. The conjugated bile acids form further complexes with sodium to become bile salts. Several steps of the pathways biosynthesis of bile acids from cholesterol are shown Figure 2. In humans, the concentration of bile salts in gallbladder can reach to 14 mM [21]. The concentrations of bile salts in different body compartments are shown in Table 2 [22]. Bile salts are categorized into three groups according to their conjugation with amino acids and their degree of hydroxylation, including trihydroxy conjugates (such as taurocholate and glycocholate), dihydroxy conjugates (such as glycodeoxycholate, glycochenodeoxycholate, taurochenodeoxycholate and taurodeoxycholate) and unconjugated forms (such as cholate, deoxycholate, and chenodeoxycholate) [23]. Approximately 60% of the bile salts in human bile are dihydroxy [24]. The dihydroxy and trihydroxy bile salts enable the solubilization of insoluble lipids including phospholipids, monoglycerides and long chain alkyl alcohols [25].

**Table 1:** Various types of bile acids [9].

Bile Acid	Abbreviation	R1(C-3)	R2(C-6)	R3(C-7)	R4(C-12)	R5(C-24)
Glycocholate	GC	OH (α)	H	OH (α)	OH (α)	NHCH_2_COO^−^
Taurocholate	TC	OH (α)	H	OH (α)	OH (α)	NHCH_2_CH2SO^−^_3_
Glycolithocholate	GLC	OH (α)	H	H	H	NHCH_2_COO^−^
Glycohyocholate	GHC	OH (α)	OH (α)	OH (α)	H	NHCH_2_COO^−^
Tauroursodeoxycholate	TUDC	OH (α)	H	OH (β)	H	NHCH_2_CH_2_SO^−^_3_
Taurohyodeoxycholate	THDC	OH (α)	OH (α)	H	H	NHCH_2_CH_2_SO^−^_3_
Glycohyodeoxycholate	GHDC	OH (α)	OH (α)	H	H	NHCH_2_COO^−^
Glycochenodeoxycholate	GCDC	OH (α)	H	OH (α)	H	NHCH_2_COO^−^
Glyco-7-oxo-lithocholate	G-7-oxo-LC	OH (α)	H	=O	OH (α)	NHCH_2_COO^−^
Taurodeoxycholate	TDC	OH (α)	H	H	OH (α)	NHCH_2_CH_2_SO^−^_3_
Taurochenodeoxycholate	TCDC	OH (α)	H	OH (α)	H	NHCH_2_CH_2_SO^−^_3_
Glycodeoxychoate	GDC	OH (α)	H	H	OH (α)	NHCH_2_COO^−^
Glycoursodeoxycholate	GUDC	OH (α)	H	OH (β)	H	NHCH_2_COO^−^
Taurolithocholate	TLC	OH (α)	H	H	H	NHCH_2_CH_2_SO^−^_3_
Taurohyocholate	THC	OH (α)	OH (α)	OH (α)	H	NHCH_2_CH_2_SO^−^_3_
Glycol-3α-6-keto-5β-cholate	Glycol3α6keto-5β-cholate	OH (α)	=O	H	OH (α)	NHCH_2_COO^−^
Tauro-α-hyocholate	T-α-MC	OH (α)	OH (α)	OH (α)	H	NHCH_2_CH_2_SO^−^_3_
Tauro-β-hyocholate	T-β-MC	OH (α)	OH (α)	OH (β)	H	NHCH_2_CH_2_SO^−^_3_

α, indicates a steric orientation below the steroid ring plane and β above.

**Table 2:** The concentrations of bile salts in human body [22].

Compartment	Concentration
Gall bladder	10–50 mmol/L
Gut	~4–20 mmol/L
Liver Canaliculi	~5 mmol/L
Portal vein blood	0.1 mmol/L
Peripheral blood	5–20 μmol/L

Bile acids perform a negative feedback regulation on their synthesis. The binding of excess bile acids by the nuclear receptor farnesoid X receptor (FXR) induces the expression of small heterodimer partner (SHP), transcriptional repressor. Then SHP interact with other transcription factors, liver receptor homolog-1 (LRH-1) and hepatocyte nuclear factor-4α(HNF-4α), that bind to the bile acid response elements (BAREs) located within the promoter region of the 7α-hydroxylase and 12α-hydroxylase genes, which results in repression of bile acid synthesis [16,26].

## 3. Physiological Functions

The main biological function of bile salts is to solubilize dietary lipids and liposoluble vitamins in the gut, transport lipids, enhance proteolytic cleavage of dietary proteins and accelerate their absorption [4,27,28,29]. Bile acids induce biliary lipid secretion and due to their physico-chemical properties can form mixed micelles with phospholipids which assist in cholesterol solubilization in the gallbladder, as a result, prevent formation of cholesterol gallstones [16,30]. While some studies demonstrated that the micelles formed by bile salts play a role in the early crystallization that leads to gall stone formation in human [28], it is known that long term treatment of human gallstone patients with chenodeoxycholic acid (CDCA) decreases hepatic very-low-density lipoprotein (VLDL) production and plasma triglyceride levels [31]. Results showed that conjugated bile salts have superior emulsification properties compared to unconjugated bile salts and can better facilitate the absorption of lipophilic compounds [9]. About 95% of bile acids are reabsorbed in the ileum and transported to the liver to inhibit 7α-hydroxylase and bile acid synthesis. 7α-hydroxylase deficiency leads to hypercholesterolemia and atherosclerosis in humans [31]. Also, they possess potent antimicrobial activity in the intestine [4] especially, against Gram-positive bacteria. The antimicrobial activity of bile salts may be related to oxidative DNA damage [32], disrupting cell membranes and cellular homeostasis [33]. It was observed that cirrhotic patients, who secrete significantly lower amounts of bile salts than healthy people, contain higher levels of bacteria in the intestine. The patients often affected by systemic infections [32]. They can modulate secretion of lipoproteins from hepatocytes [15] and at low concentrations, stimulate proliferation of the colonic epithelium and are involved in the regulation of colonic mucosal growth [34]. Bile acids can influence on immune cells in the mucosa [35] and stimulate intestinal immunity [36]. As signaling molecules, bile acids are able to modulate glucose, lipid and energy metabolism [30]. They stimulate activation of G protein coupled receptor (TGR5) in the enteroendocrine cells that promote glucagon like peptide-1 (GLP-1) release. GLP-1 is important mediator of insulin release from pancreatic β cells [37]. It was observed that bile acids are able to induce activation of nicotinamide adenine dinucleotide phosphate oxidase in hepatocytes, and the following increase reactive oxygen species (ROS) production that is essential for bile acid induced apoptosis [29]. In addition, bile acids facilitate intestinal calcium absorption [16].

Bile salts are secreted into the duodenum and adsorb onto the surface of lipid to further emulsify them and to prepare this interface for the enzymatic break down by the pancreatic lipases. Lipases can hydrolyses the lipids that composed of triglycerides, into free fatty acids, monoglycerides and diglycerides. Some of these compounds are soluble, therefore they can be removed from the surface of lipid and become incorporated within micelles of bile salts in order to be absorbed by the intestinal mucosa [38]. Bile salts are capable to inhibit the precipitation of furosemide drug solutions owing to its pH-dependent solubility [39]. Studies showed that bile acids play key homeostatic roles in glucose metabolism, xenobiotic detoxification of toxins, cholesterol and lipid metabolism. Moreover, they can employ for the treatment of illnesses such as tamoxifen-resistant breast cancer, prostate cancer, colon cancer, Alzheimer’s disease, atherosclerosis, obesity and metabolic disorders [40].

## 4. Physico-Chemical Properties

Because of several physiological functions of bile salts, their micellar properties have been extensively studied. In aqueous solutions, bile salts aggregate and form micelles in concentrations above critical micelle concentration (CMC) [11]. By forming micelles, bile salts can facilitate transcellular passage and enhance absorption [41]. CMC values of some bile salts are mentioned in Table 3. CMC values are determined by surface tension and dye solubilization methods [12]. Surface tension is measured by an improved maximum bubble pressure method. This technique measures the pressure in a bubble formed at the end of an immersed capillary into which flows a constant stream of air. The bubble reaches its maximum pressure when its diameter is equal to that of the capillary, which occurs just before the bubble escapes. The maximum pressure is proportional to the surface tension. The surface tension depends on the monomer activity in solution. By increasing concentrations, aggregation begins to form and the surface tension *versus log* concentration graph shows a significant reduction in slope in the CMC region [12]. Dye solubilization method is performed using Orange OT (1-*O*-tolyl azo-2-naphthol), which is a water-insoluble and micelle-soluble dye. Solubilization of Orange OT takes place only when micelles are present, and the solubilized amount is directly proportional to the concentration of micelles [42]. Also, the fluorescence probe technique is used for determination of CMC. This method involves the use of hydrophobic fluorescence dyes (such as pyrene and pyrene-3-carboxaldehyde) which are sensitive to the polarity of the solubilizing medium and will exhibit different fluorescence behavior in micellar and nonmicellar solutions. Such changes of behavior as a function of surfactant concentration have been used to determine the CMC. Ananthapadmanabhan *et al.* in 1985 showed that the CMC values estimated from the fluorescence characteristics of pyrene-3-carboxaldehyde was consistently closer to CMC values of surfactants that determined using surface tension [43].

Bile salts can form either primary or secondary micelles. Primary bile salt micelles have aggregation numbers from 2 to 10 and are formed via hydrophobic interactions, while secondary micelles (aggregation numbers 10–100) are formed via hydrogen bonding interactions of the primary micellar structures (Figure 2) [18,44]. The pH, at which CMC formation occurs, is called the critical micellar pH (CMpH) [13] at which the solubility increases markedly [45]. CMC of dihydroxy bile salts are usually below CMC of trihydroxy bile salts. The high CMC of trihydroxy bile salts is ascribed to their higher solubility in water [18]. It has been reported that conjugation with glycine or taurine slightly lowers CMC of trihydroxy bile salts [46]. The orientation of hydroxy substituents also influences the CMC values and changing of a hydroxy substituent from α-to β-configuration increases the CMC values. Moreover, addition of Na^+^ ion to a total concentration of 0.15 M lowers CMC [12].

**Table 3:** CMC value of some bile salts.

Bile Salt	CMC (mM)	Ref.
NaTC	8	[47]
NaC	4	[48]
NaLC	1	[45]
NaGC	2–5	[48]
NaTCDC	2.5–3	[49]

GDC: Glycodeoxychoate; NaTC: sodium taurocholate; NaC: sodium cholate; NaLC: sodium lithocholate; NaGC: sodium glycocholate; NaTCDC, sodium taurochenodeoxycholate.

## 5. Bile Salts as Absorption Enhancer

Because of biocompatibility, bile salts have been widely used as permeation enhancers. They can enhance drug penetration through various biological membranes by interacting with phospholipids in cell membranes. Therefore, they have been employed as permeation enhancers in topical dosage forms including buccal, ocular, nasal, and transdermal routes of administration [39,50].

### 5.1. Oral Drug Delivery

**Figure 3:**
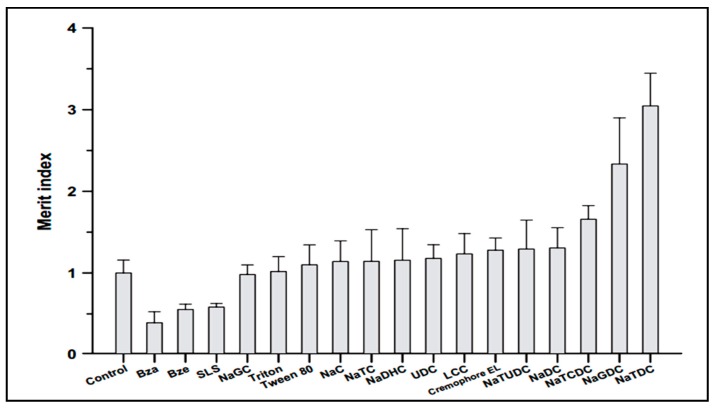
Effect of various surfactants on the Merit index of sCT.SLS: sodium lauryl sulfate, Bza: benzalkonium chloride, Bze: benzetonium chloride, NaGC: sodium glycocholate, NaC: sodium cholate, NaTC: sodium taurocholate, NaDHC: sodium dehydrocholate, UDC: ursodeoxycholate, LCC: lauroylcarnitine chloride, NaTUDC: sodium tauroursodeoxycholate, NaDC: sodium deoxycholate, NaTCDC: sodium taurochenodeoxycholate, NaGDC: sodium glycodeoxycholate, NaTDC: sodium taurodeoxycholate [53].

Drug administration through the oral route is the most preferred route by patients and has many advantages including, children’s acceptabilty, ease of administration, lack of pain and discomfort associated with injections [51,52]. Song *et al.* investigated enhancement of intestinal absorption of salmon calcitonin (sCT) from proliposomes containing bile salts [53]. Oral bioavailability of sCT is very low due to enzymatic degradation in gastrointestinal tractand poor permeation across intestinal epithelial cells. One approach for overcoming this problem is the use of absorption enhancers. Song *et al.* applied different permeation enhancers to improve intestinal absorption of sCT. According to Figure 3, in comparison with different absorption enhancers, sodium taurodeoxycholate (NaTDC) (drug:NaTDC; 0.75:2.5) showed the largest Merit index value. The Merit index was calculated as the ratio of the fold increase in permeability of sCT over the fold decrease in transepithelial electrical resistance (TEER) value in Caco-2 cell monolayers for each compound. It has been previously reported that some of bile salts including NaTDC, are known to form lipophilic ion-pair complexes with various organic cations, which increasethe permeability of the cations across biological membranes. Since sCT is a hydrophilic and cationic molecule, it was demonstrated that the effect of NaTDC in enhancing permeability of sCT might be due to ion-pair forming ability of the bile salts with sCT [53].The findings of Song *et al.*, is contrary to the results of Cetin *et al* [54], that reported no significant difference was found between nanoparticle formulations with and without NaTDC on oral absorption of sCT (drug:NaTDC; 1:1) [54]. This difference might be due to the used ratio of drug:NaTDC, which were quality different.

It has been previously shown that sodium glycocholate (NaGC) could improve insulin absorption more than sodium salicylate as absorption enhancer through oral delivery [55]. It was observed that the effect of NaGC on intestinal absorption of insulin is site dependent and the duodenum appeared to be the optimal site for insulin oral delivery [56].

### 5.2. Transdermal Delivery

The major obstacle for drug transportation through skin is the outer layer of skin, the stratum corneum. It is the actual physical barrier to most substances contacting skin [57,58,59]. Many approaches have been employed in order to increase absorption of drugs through skin. Among them is utilization of absorption enhancers such as bile salts. Sodium deoxycholate (NaDOC) is one of the bile salts that forms stable gels and can be useful as drug carrier for topical skin application [60]. Senyigit *et al.* in 2011, observed that *in vitro* flux of betamethasone-17-valerate from NaDOC gels across rat skin was 2.5 (0.05%) and 8.5 times (0.1%) higher than commercial cream (0.1%), respectively. Furthermore, *in vivo* anti-inflammatory activity was in agreement with *in vitro* drug permeation. Histology studies indicated that NaDOC gel has no irritant effect on the skin [61]. It has been also shown that sodium tauroglycocholate (NaTGC) is a potent absorption enhancer for drug delivery to the skin. Kouchak *et al*. in 2014 found that NaTGC (100 μg/mL) improved the flux of aminophylline through snake skin compared to simple gel (*p* < 0.05) [62]. Moreover, in previous studies, it was reported that NaTGC could increase absorption of theophylline through shed snake skin [63]. It has been suggested that NaTGC, as a surfactant, enhances the penetration of compounds into stratum corneum followed by interaction with keratin filaments that leads to corneocyte disruption or may modify peptide or protein in the lipid bilayer stratum corneum that increase permeability [64].

### 5.3. Nasal Delivery

Nasal drug delivery is a suitable route for low dose drugs which show poor stability in the gastrointestinal tract. Drugs administered intranasally avoid liver first-pass metabolism and the route is non-invasive, making ease of administration by the patient in long term therapy a favorable aspect of nasal delivery [65,66]. The most important factor limiting the nasal absorption of polar drugs is low membrane permeability [67]. To overcome this problem, different approaches have been attempted, including the use of absorption enhancers such as bile salts [68,69]. As shown in Table 4, many researchers have investigated bile salts as absorption enhancers for nasal delivery of insulin. Hirai *et al.* in 1981 demonstrated that bile salts may promote insulin transport across nasal mucosa by retarding insulin degradation by leucine aminopeptidase, a proteolytic enzyme. These compounds were found to be less irritating to the nasal mucosa, have lower hemolytic activity and less protein release than other surfactants [70]. In addition, it has been reported that bile salts may affect the nasal membrane by creating temporal pores and enhance the permeability of it, or form reverse micelles within nasal membranes, which insulin monomers can diffuse through polar channels from the nares into the blood stream [71]. It has been shown that bile salts cause nasal irritation when used above a concentration of 0.3% [72]. Sodium glycodeoxycholate (NaGDC) is more effective as an absorption promoter in the nasal mucosa than sodium glycocholate (NaGC). Because of the lack of a hydroxyl group at the 7 position of the steroids, NaGDC is more lipophilic than NaGC [73]. Nevertheless, NaGC has been widely used as an enhancer in nasal absorption due to its relatively low toxicity compared to other bile salts. It is believed that NaGC is able to transport large and hydrophilic molecules by interaction with membrane lipids, enzymatic inhibition and opening of tight junctions between epithelial cells [74].

**Table 4:** Bile salts as absorption enhancers for nasal delivery of insulin.

Bile Salts	Animals	Result	Ref.
Sodium deoxycholate	Rat	Blood glucose levels dropped to 60% of initial levels after 30 min	[75]
Sodium glycocholate	Rat	Increases insulin efficacy	[76]
Sodium taurocholate	Rabbit	Microcrystalline cellulose suspension containing insulin and sodium taurocholate (1% *w*/*w*) sprayed into the nasal cavity provided a bioavailability of 8.36%	[77]
Sodium glycocholate	Dog	Increases absorption of insulin	[78]
Sodium cholate	Rabbit	Reduces blood glucose (60.06%)	[79]

### 5.4. Buccaland Mucosal Drug Delivery

Buccal drug delivery has high patient acceptability due to the elimination of pain associated with injections. It is fully vascularized and more accessible for the administration and removal of a dosage form. Nausea and vomiting induced through oral administration is avoided. Furthermore, drugs, which show poor bioavailability *via* oral route, can be administered conveniently through buccal route. But, buccal mucosa is less permeable and so unable to give a rapid absorption window. Penetration enhancers are capable of decreasing penetration barrier of buccal mucosa [80]. Bile salts have been extensively investigated for their ability to enhance buccal penetration of tracer molecules and drugs [81]. SDGC is widely used to improve the transbuccal delivery of drugs [82]. Hoogstraate *et al.* in 1996 showed that enhancement effect of SGDC on buccal permeation is concentration dependent. They observed that SDGC at concentration of 10 mM enhanced the flux of fluorescein isothiocyanate (FITC)-dextran through paracellular routes, while, at high concentrations (100 mM) enhanced drug delivery through paracellular and transcellular routes [83]. Sodium cholate is present in the RapidMist^™^ spray that is used for delivery of insulin to the buccal mucosa [84]. Regarding the differential scanning calorimetry (DSC) results of Gandhi *et al.*, 1992, it was demonstrated that the influence of sodium deoxycholate on transbuccal delivery of salicylic acid may be related to the effect of penetration enhancer on the protein domain that involve the uncoiling and extending of the protein helix, and thereby opening the polar pathway [85]. It has been found that unconjugated bile salts that are less hydrophobic, are more effective than their conjugated forms in absorption of calcitonin through buccal rat mucosa *in vivo* [86]. Oral administration of decitabine (an anticancer drug) is not ideal because of its degradation under the acidic conditions in the stomach. Also, due to poor chemical stability of decitabine, intravenous infusion is not appropriate. In 2007 Mahalingam *et al.*, evaluated the feasibility of transbuccal delivery of decitabine and the effect of NaTC, NaGC, sodium deoxytaurocholate (NaDTC) and sodium deoxyglycocholate (NaDGC) on its permeability. The permeation enhancement of dihydroxy bile salts (NaDTC and NaDGC) across the buccal mucosa was better than that of trihydroxy bile salts (NaTC and NaGC). Furthermore, it was observed a 38-fold enhancement in flux was achieved with 10 mM of NaDGC. These researchers were believed that the enhancements in the flux of decitabine in the presence of bile salts may occur by a complex process including solubilization and micellar entrapment of intercellular lipids, denaturation and extraction of proteins, enzyme inactivation and tissue swelling [50].

### 5.5. Rectal Drug Delivery

**Figure 4:**
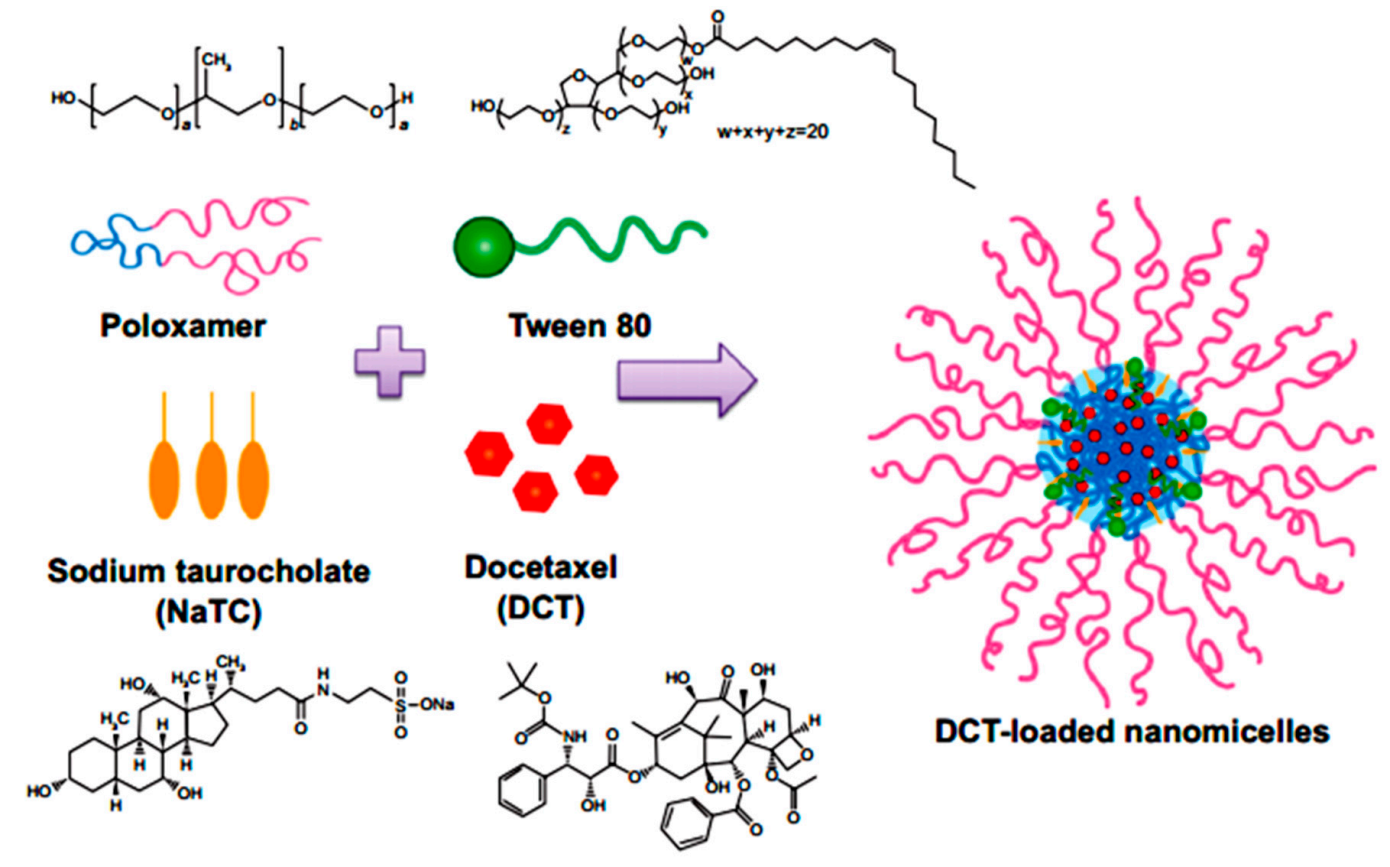
Preparation of DCT-loaded nanomicelles containing NaTC [88].

**Figure 5:**
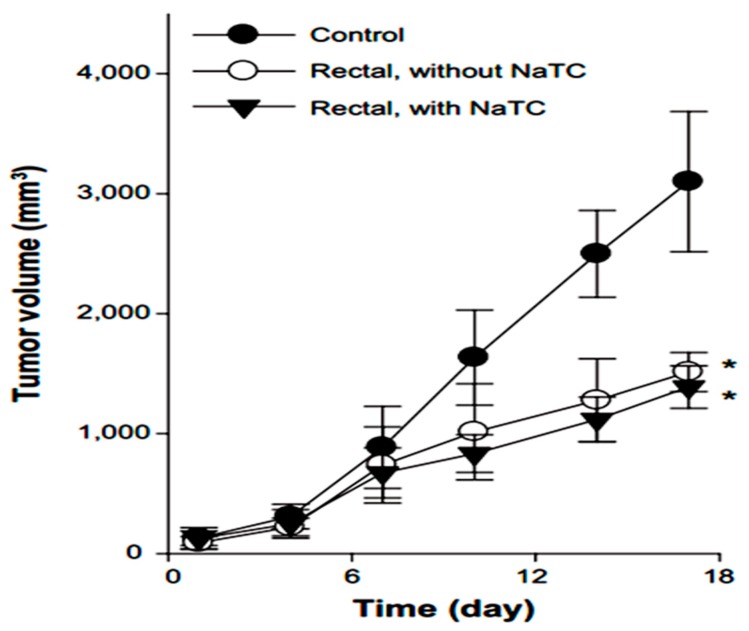
Anti-tumor efficacy of DCT-loaded nanomicelles with and without NaTC after rectal administration [88].* Significantly smaller than control group (*p* < 0.01).

Yamamoto *et al.* in 1992 investigated the effect of NaGC, NaTC and sodium deoxycholate (NaDC) on the improvement of rectal penetration of insulin in the albino rabbit. They found that NaGC was more effective than NaTC but less effective than NaDC in improving the rectal absorption of insulin in rabbits [87]. Docetaxel (DCT) is one of the most anti-cancer drugs in treatment of head and neck cancer, breast cancer, gastric cancer and ovarian cancer. Due to low aqueous solubility and poor oral bioavailability, DCT has greatly limited therapeutic applications. Therefore, Kim *et al.* in 2014 designed nanomicelles as delivery systems composed of DCT/poloxamer 407 (P407)/poloxamer 188 (P188) Tween 80/NaTC for improvement of the bioavailability and anti-tumor efficacy of DCT upon rectal administration (Figure 4). Poloxamer, was selected for the formulation of unique thermosensitive and bioadhesive DCT-loaded nanomicelles. Because its reverse gelation property, polaxamer remains in liquid state at room temperature (~25 °C), while gelifying at physiological body temperature (~37 °C). Also, Tween 80 acts as an additional solubilizing agent, and can form a eutectic mixture with DCT. As shown in Figure 5, compared to control, DCT-loaded nanomicelles significantly reduced the tumor growth of mice (*p* < 0.001). Moreover, the pharmacokinetic results showed that NaTC was influential in improving the half-life and plasma level of DCT. Although the elevated plasma level of DCT from the bile salt group did not enhance the anti-tumor potential, DCT-loaded nanomicelles could reduce the drug-related side effects, such as hypersensitivity reactions and fluid retention, while retaining the potential anti-tumor efficacy in clinical subjects [88]. 

### 5.6. Ocular Drug Delivery

Saettone *et al.* in 1996 evaluated bile salts as absorption enhancers for permeation of β-blocking agents (atenolol, timolol, levobunolol and betaxolol) through isolated rabbit corneas. According to their results, the permeation of atenolol was significantly increased (5.8-fold) by 0.05% TDC. Also, DC and UDC at concentrations 0.05% could enhance permeation of timolol about 5.2 and 2.1-fold, respectively [89]. Yamamoto *et al.* in 1989 investigated systemic insulin delivery in the albino rabbit with NaGC, NaTC, NaDC at a concentration of 1%. The results showed bioavailability of insulin was 4.9% to 7.9% with NaGC, 3.6% to 7.8% with NaTC and 8.2% to 8.3% with NaDC, as compared to 0.7% to 1.3% in the absence of absorption promoters [90].

## 6. Mechanism of Action of Bile Salts as Absorption Enhancers

**Figure 6:**
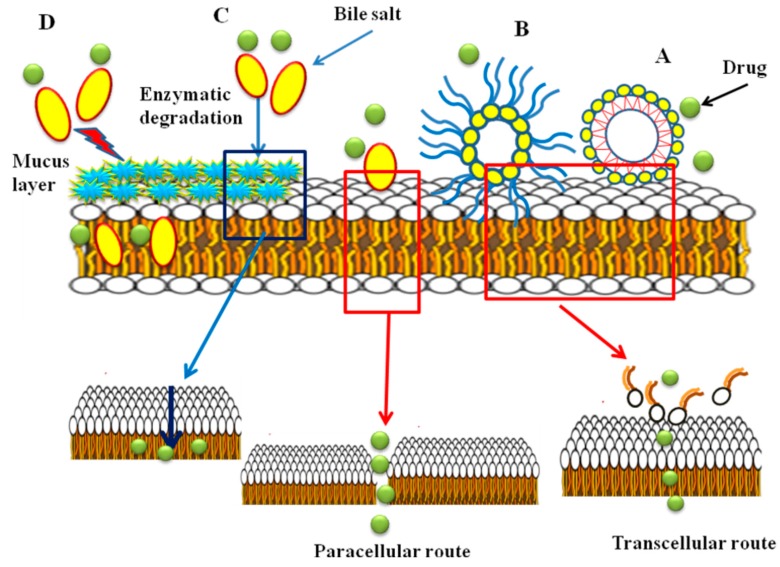
The most common transport mechanisms of bile salts; (**A**) micelle formation; (**B**) reverse micellization; (**C**) enzyme inhibition; (**D**) viscosity reduction of the mucus layer.

The mechanism of absorption enhancement by bile salts includes extracting membrane protein or lipids, membrane fluidization, producing reverse micelles in the membrane and creation aqueous channels. It is reported that at higher concentrations of bile salts, membrane lipids may be extracted, to form micelles and enhance transcellular transport [41,80,91]. Bile salts enhance transport of hydrophilic drugs through the paracellular route by incorporation into the cell membrane where, at a certain concentration, they could form reverse micelles that included water molecules, thus creating hydrophilic pores in the cell membrane [92]. Also, the bile salts can increase the paracellular transport by disruption of the hemidesmosomes [93] or by binding to Ca^+2^ in the regions of tight junctions [94,95]. It is believed that filamentous actin (F-actin) play a major role in controlling of paracellular permeability. According to the results of F-actin staining studies and reduction of transepithelial electrical resistance (TEER) values in the presence of bile salts performed by Lin *et al.*, damage to the tight junction between the epithelial cells and thus an increase in paracellular permeability was reported [96]. Moreover, bile salts can reduce the viscosity and elasticity of the mucus layer adhering to all mucosal surfaces and consequently increase epithelial membrane permeability [95,97,98]. In different studies, it has been shown that bile salts have inhibitory effects on mucosal membrane peptidases [6]. The effect is reversible and concentration dependent [99]. It was found that sodium glycocholate acts as a protease inhibitor, to reduce insulin metabolism on mucosal membranes 5-fold [100]. In addition, it was reported that the effect of bile salts on the oral mucosal absorption of human calcitonin in rats was related to the inhibition of degradation of calcitonin in mucosa [101]. It was also observed that the effect of bile salts on the transport of drugs is concentration dependent. Senel *et al.* in 1998 reported that enhancement of the permeation of morphine hydrochloride through buccal epithelium was dependent on the concentration of sodium glycocholate (NaGC). Drug permeation was increased in the presence of 100 mM NaGC, whereas no enhancement was obtained at 10 mM NaGC [48]. It has been reported that some bile salts inhibit the active efflux of P-glycoprotein (Pgp) substrates, probably indirectly, by changing the lipid environment of the Pgp transporter or by interaction with Pgp. Studies showed that Pgp function was not affected by CA, DC and TC, whereas taurolithocholate (TLCA), taurochenodeoxycholate (TCDC), glycochenodeoxycholate (GCDC) and UDC inhibit Pgp mediated drug transport. This may be due to the absence of a hydroxyl group at position 12 in TLCA, TCDC, GCDC and UDC that determines the structural property of inhibiting Pgp [92]. The most commonly known transport mechanisms of bile salts are shown in Figure 6.

## 7. Toxicity

**Figure 7:**
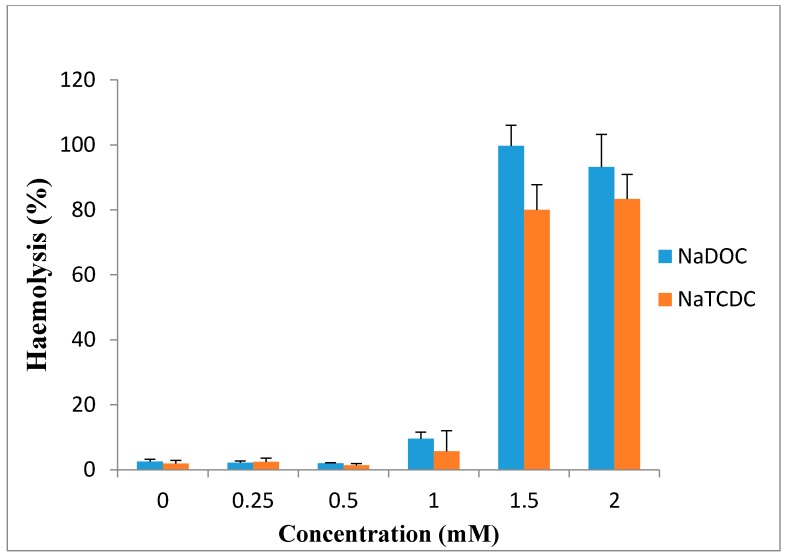
Percentage haemolysis of red blood cells caused by incubation with differing concentrations of NaDOC and NaTCDC (mean ± SE, *n* = 8) (data are taken from [106]).

Bile salts have limited clinical use because of the irreversible damage to the mucosa and ciliotoxicity. It has been reported that dihydroxy bile salts are more toxic than trihydroxy bile salts. Studies showed deoxycholate is intensively ciliotoxic, with ciliary arrest occurring within 1 min at a concentration of 5 mM [97]. These findings are in accord with the observation of Guus *et al*. in 1986, who reported that deoxycholate is extremely ciliotoxic at a concentration of 5 mM when used for intranasal absorption of gentamicin [102], while ciliotoxicity of taurocholate is less and ciliary arrest does not occur until 30 min, even at concentration of 30 mM [86]. Wheatley *et al.* in 1988 observed that 1% deoxycholate solution caused irreversible tissue damage to sheep nasal mucosa [103]. Based on the study of Yang *et al.*, sodium deoxycholate enhanced morphine-6-glucuronide uptake in rat brain endothelial 4 (RBE4) cells due to a cytotoxic effect. 97% cytotoxicity and 100% cytoxicity was produced at 0.5 and 1 mM concentration, respectively [3]. Saettone *et al.* in 1996 found that among the bile salts (sodium deoxycholate, sodium taurodeoxycholate sodium ursodeoxycholate and sodium tauroursodeoxycholate), sodium deoxycholate at concentration of 1% was irritant and caused corneal damage [89]. Bowe *et al.* reported in 1997 that sodium deoxycholate is an effective absorption enhancer for insulin. In the presence of this compound, blood glucose levels decreased to 60% of initial levels after 30 min, but in comparison with the other absorption enhancers, it was somewhat irritant [75]. Also, high concentrations of sodium deoxycholate can damage plasma and nuclear membranes [104]. Morimoto *et al.* in 1998 reported that the ciliary beat activities and mucociliary transport rates were not significantly affected by sodium glycocholate at concentrations of 1, 10 and 20 mM, whereas they were significantly reduced by 1 and 10 mM sodium taurodeoxycholate and immediately stopped after application of 20 mM taurodeoxycholate. Based on these results, they concluded that sodium glycocholate may be a safe and useful absorption enhancer for intratracheal drug delivery [105]. As shown in Figure 7, 0.25, 0.5 and 1 mM sodium deoxycholate (NaDOC) and sodium taurochenodeoxycholate (NaTCDC) caused no significant change in hemolytic activity of the bile salts on the red blood cells (RBCs), while incubation at 1.5 and 2 mM concentrations, significantly increased haemolysis (*p* < 0.0002) [106].

**Figure 8:**
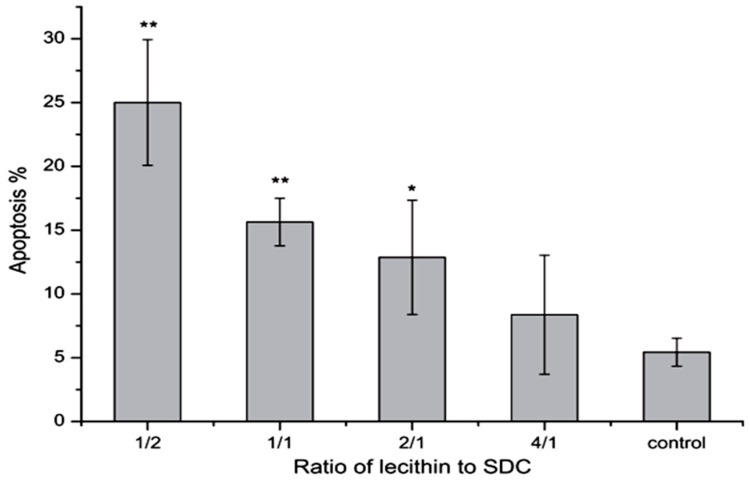
Effects of 0.2 mM mixed micelles with different lecithin/NaDC ratios on the apoptosis of Caco-2 cells (*n* = 5) [108]. * *p* < 0.05, ** *p* < 0.01.

It has been shown that phosphatidylcholine can prevent toxicity of bile salts on gastrointestinal epithelia and membrane [21]. In 2000 Barrios *et al.* stated that this effect may be related to formation of less toxic mixed micelles [107]. Besides, phosphatidylcholine as an ingredient of the bile is able to bind to bile salts and form mixed micelles that can improve the dissolution of insoluble drugs [21]. Tan *et al.* in 2013 found that mixed micelles of 0.2 mM NaDC with 1/2–2/1 of lecithin/NaDC ratios induced significant apoptosis on Caco-2 cells compared to the control group, although, micelles with 4/1 ratio of lecithin/NaDC induced less apoptosis (Figure 8). These results indicate that more lecithin in mixed micelles causes less apoptosis [108]. Moreover, based on the findings of Zhang *et al*., a combination of β-cyclodextrins with NaDC reduces the intensity of nasal ciliotoxicity of NaDC [109]. It has been reported that taurine and glycine conjugates of the bile salts are somewhat less irritating to the nasal mucosa, as they are in terms of toxicity on other membranes [71].

## 8. Novel Approaches

Novel vesicular delivery systems containing bile salts and liposomes are known as bilosomes, act as penetration enhancers [110]. The incorporation of bile salts into liposomes can stabilize the membrane against the deleterious effects of physiological bile acids in the gastrointestinal tract [111] and facilitate internalization of the particles [112]. In addition, bilosomes are produced from natural lipids that make them biocompatible [113]. Guan *et al.* in 2011 encapsulated cyclosporine A in bilosomes and observed that its oral bioavailability increased due to enhanced penetration of the bilosomes [114]. Also, Conacher *et al.* in 2001 reported that cyclosporin A-encapsulating bilosomes can promote uptake by M-cells in Peyer’s patches, and therefore increase absorption through the lymphatic system [115]. Studies showed that these carriers are suitable for oral vaccine delivery [111] and may have inherent adjuvant properties [113]. Mann *et al.* in 2004 found that A/Panama influenza haemagglutinin-containing bilosomes were capable of inducing systemic and mucosal immune responses by IgG and IgA antibodies, respectively [113]. In 2006 Mann *et al.* also showed that entrapment of tetanus toxoid in bilosomes could protect the protein against harsh environment of the stomach and its oral administration induced both systemic and mucosal immunity against bacterial protein antigen [116]. The results of a Shukla *et al.* study indicated that hepatitis B surface antigen-loaded bilosomes produced both systemic and mucosal antibody responses upon oral administration. Moreover, the carriers with a fivefold higher dose upon oral administration produced comparable serum antibody titres to those obtained after intramuscular immunization without, causing systemic tolerance [117].

## 9. Conclusions

Bile salt synthesis is the major route for cholesterol elimination from the body. Bile salts have been especially considered due to their efficacy in the solubilization and improving the absorption of drugs. They have been widely explored for this purpose. Bile salts increase drug absorption via paracellular and transcellular routes, alteration of mucus layers or enzymatic degradation. Bile salts are highly important for drug absorption and they may be considered useful aids in drug formulation.
